# Chimeric Antigen Receptor-Engineered T Cell Therapy for the Management of Patients with Metastatic Prostate Cancer: A Comprehensive Review

**DOI:** 10.3390/ijms22020640

**Published:** 2021-01-11

**Authors:** Young Dong Yu, Tae Jin Kim

**Affiliations:** Department of Urology, CHA Bundang Medical Center, College of Medicine, CHA University, Seongnam 13497, Korea; a183046@chamc.co.kr

**Keywords:** CAR-T, immunotherapy, prostate cancer, T cells, metastasis

## Abstract

Prostate cancer (PCa) has a vast clinical spectrum from the hormone-sensitive setting to castration-resistant metastatic disease. Thus, chemotherapy regimens and the administration of androgen receptor axis-targeted (ARAT) agents for advanced PCa have shown limited therapeutic efficacy. Scientific advances in the field of molecular medicine and technological developments over the last decade have paved the path for immunotherapy to become an essential clinical modality for the treatment of patients with metastatic PCa. However, several immunotherapeutic agents have shown poor outcomes in patients with advanced disease, possibly due to the low PCa mutational burden. Adoptive cellular approaches utilizing chimeric antigen receptor T cells (CAR-T) targeting cancer-specific antigens would be a solution for circumventing the immune tolerance mechanisms. The immunotherapeutic regimen of CAR-T cell therapy has shown potential in the eradication of hematologic malignancies, and current clinical objectives maintain the equivalent efficacy in the treatment of solid tumors, including PCa. This review will explore the current modalities of CAR-T therapy in the disease spectrum of PCa while describing key limitations of this immunotherapeutic approach and discuss future directions in the application of immunotherapy for the treatment of metastatic PCa and patients with advanced disease.

## 1. Introduction

Estimates have shown that the mortality rate of metastatic castration-resistant prostate cancer (mCRPC) and the fatal manifestation of the disease are approximately 31,000 cases annually [1]. Currently, chemotherapy with taxanes, androgen receptor axis-targeted (ARAT) agents, and immunotherapeutic approaches using sipuleucel-T and radium-223 are Food and Drug Administration-accepted therapeutic modalities that improve overall survival (OS) in men with mCRPC [2]. Significant advances and innovation in the systematic regimens and treatment protocols for mCRPC have existed since the approval of radium-223 in 2013. Immunotherapy with checkpoint inhibitors and the use of poly (ADP-ribose) polymerase inhibitors have profoundly influenced the treatment paradigm of other solid cancers [3,4]. Immunotherapy approaches targeting prostate-specific membrane antigen (PSMA), while specific to prostate cancer (PCa), may utilize the same therapeutic mechanisms relevant to other malignancies. Continued research in the field of immunotherapy will considerably alter the treatment landscape and therapeutic approaches in mCRPC and the outcomes of patients with PCa.

Recent advances in the field of immunotherapy include adoptive cellular therapy (ACT), in which the T cells from the peripheral blood are collected through leukapheresis and consequent apheresis before being genetically modified ex vivo before reinfusion [5]. Different ACT modalities, such as tumor-infiltrating lymphocytes (TILs), engineered T cell receptors (TCR), and chimeric antigen receptor T cell (CAR-T) therapy, have been engineered and are being evaluated in various scientific studies and clinical settings [5]. Clinical results from studies analyzing the effects of CAR-T therapy in solid tumors including PCa have shown promise. In the PCa treatment setting, CAR-T cells targeting PSMA have displayed encouraging results, suggesting a potential translational treatment target for the treatment of PCa in the advanced clinical setting [6]. Collectively, these clinical outcomes will open new horizons in the treatment and cure for the disease continuum of PCa. This article provides a thorough overview of the role of CAR-T cell therapy in advanced PCa and the metastatic setting will be provided along with an extensive review of current and future modalities that will further improve the therapeutic spectrum in the ongoing treatment of advanced PCa and metastatic disease.

## 2. The Molecular Construct of CAR-T Cells

CAR-T cells are constructed on the molecular level in which the effector portion of the T lymphocytes recognizes and attacks the cells bearing the specific surface target antigen. This interaction occurs in an environment that does not require the immunologic action of antigen-presenting cells (APC), unlike the molecular signaling required for unmodified T cell activation. Target antigen recognition and the intracellular signaling cascade in CAR-T cells are facilitated by the CAR molecule, which incorporates the recognition of the target antigen along with transmembrane and intracellular signaling. The major characteristic that distinguishes each generation of CARs is the intracellular presence of one or more costimulatory domains in the CAR. Interestingly, this variation in the costimulatory structure determines the immunologic function, molecular phenotype, and proliferative capacity of CAR-T cells and the current preferred immunotherapeutic modality in the second- or third-generation CAR layouts [7]. Moreover, CAR molecules consist of three individual components. The extracellular domain, which identifies the target antigen identification, has a single-chain fragment variable (scFv) structure that binds with tumor-associated antigens (TAA). The attachment of scFv to the T cell is possible due to the transmembrane domain, which comprises of the transmembrane regions CD 3, CD 8, CD 28, or FcεRI. The transmembrane sector is linked to the intracellular zone situated in the intracytoplasmic domains of CD 8, CD 28, CD 137, and CD 3ζ. The intracellular zone encompasses the immune receptor tyrosine-based activation motif (ITAM) that plays an essential role in signal transduction for T cell activation [8].

**Figure 1:**
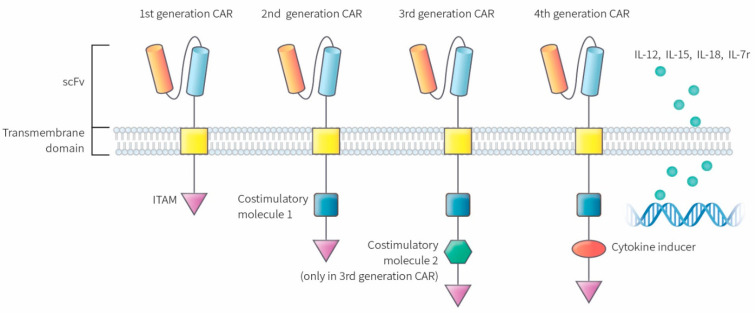
Molecular structure of different CAR generations. The first generation CAR only contains ITAM motifs in the intracellular domain. Second-generation CARs included the addition of one co-stimulatory molecule and third-generation CARs contain a second co-stimulatory molecule. The fourth generation of CARs was based on second-generation CAR construct paired with a constitutive cytokine inducer. These types of CAR-T cells are also known as TRUCKs. CAR: chimeric antigen receptor, scFv: single-chain fragment variable, ITAM: immunoreceptor tyrosine based activation motifs, TRUCK: T cell redirected for universal cytokine-mediated killing.

CARs are classified into four different classes depending on the molecular structure and complexity (Figure 1). The first-generation CAR consists of a simple receptor divided into the aforementioned components (scFv, a transmembrane domain, and intracellular zone). The first-generation CAR-T cells failed to obtain a significant number of activated T lymphocytes in blood circulation with this molecular arrangement, although enabling T cell activation was possible due to the absence of a costimulatory molecule [9,10,11]. The second-generation CAR was engineered to overcome this clinical limitation by additionally inserting a costimulatory protein (CD 28, CD 27, CD 134, or CD B7) into the intracellular domain. Additional costimulatory molecular structures such as CD 28, 4-1BB, and CD 3ζ were inserted during the development of the third-generation CAR to increase the concentration of activated T cells in the circulatory system [12]. The T cells redirected for universal cytokine-mediated killing (TRUCKs), which has a costimulatory domain and pro-inflammatory construct, such as interleukin (IL)-12, which increases the effectiveness of circulating T cells, are the fourth iteration of these molecules [13]. IL-12 aims to mitigate the immunosuppressive properties of the tumor microenvironment (TME) by activating the T helper 1-type cell cascade [14,15]. However, additional modifications to the fourth-generation CARs are not restricted to IL-12 because various categories of molecules have been engineered or modified to use in TRUCK construction. Other molecular combinations consist of cytokines, such as IL-15 to developmentally enhance the T memory stem cells [16] and IL-18 [17] along with cytokine receptors, which include the IL-7 receptor (C7R), to reduce the adverse effects of cytokine toxicity [18]. Apart from the aforementioned molecules, the knock-out genes, including PD-1 or diacylglycerol kinase, and knock-in genes, such as TCRα subunit constant gene or chemokine C-X-C motif receptor (CXCR) 4, are included in the TRUCK cassette to improve CAR expression and its antitumor properties [19,20]. Therefore, controlled and inducible systems (Syn/Notch) and antigen combinations of human epidermal growth factor receptor 2 (HER 2) + IL-13 receptor subunit α2 (IL-13Rα2) have been synthesized and utilized to mediate effective expression and prevent antigen escape [21].

## 3. The CAR-T Cell Therapy Platform

**Figure 2:**
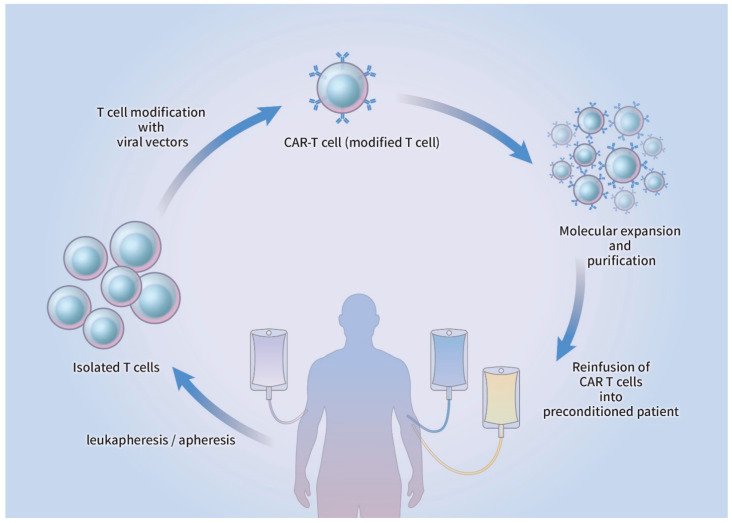
Schematic drawing of CAR-T cell production. T cells from peripheral blood are isolated via leukapheresis, followed by apheresis. The T cells are transduced by viral/non-viral vectors and genetically modified to express a CAR. After ex vivo expansion and purification, CAR-T cells are reinfused into the patient who received prior lymphodepletion therapy. CAR-T: chimeric antigen receptor T cell.

Initiation of CAR-T cell therapy commences with the harvesting of lymphocytes from the peripheral blood by leukapheresis, which subsequently undergoes apheresis without the additional insertion of granulocyte colony-stimulating factor (G-CSF) [22]. The T cells are genetically refined and infected with a viral vector that can be retroviral or lentiviral in origin or non-viral CAR vector, where genomic DNA is artificially inserted. The modified CAR-T cells are introduced back into the blood circulation of the patient, who usually undergoes lymphodepletion therapy before CAR-T reinfusion, after molecular expansion and purification ex vivo [22,23]. The CAR-T cells become activated after the infused CAR interacts with the antigen presented on the surface of the target cell, which causes lysis of the target cell along with the production and proliferation of cytokines (Figure 2).

The molecular structure and physical properties of the receptor, transmembrane domains, and signaling zones of the individual CAR significantly influence the therapeutic effects of CAR-T cells, which make the engineering of CARs essentially an experimental approach. Moreover, CAR-T cell therapy has shown promising results in the area of hematologic malignancies, where complete response (CR) was achieved in more than half of the patients with acute lymphoblastic leukemia (ALL), chronic lymphocytic leukemia (CLL), and lymphoma [24,25]. The therapeutic advantages of CAR-T cell therapy are yet to be reiterated in patients with solid cancers, including breast cancer, melanoma, and sarcoma, in contrast to hematologic cancers [26,27]. The number of cancer-specific surface antigen is scarce in patients with solid cancers, and as a consequence, the success rates of the CAR-T cell therapy in destroying tumor cells and controlling TME are low. Total recognition and high identification rates of PCa-specific surface epitopes by CARs should be of paramount concern in current and novel CAR-T cell immunotherapy regimens to be effective in the treatment of metastatic PCa [28].

## 4. The Role of Prostate Tumor Associated Antigens in CAR-T Cell Therapy

Identifying prostate TAA is the initial and crucial step for CAR-T cell therapy to be therapeutically effective. A potential target antigen should be specifically expressed by the tumor cells to elicit CAR-T cells to activate an immunologic response that reacts to its target cancer cell and thereby prevent unwanted immune reactions with healthy cells and organs [29,30]. With PCa, the expressed antigens by prostate carcinoma are prostate-specific antigen (PSA), PSMA, prostatic acid phosphatase (PAP), and prostate stem cell antigen (PSCA). Recently, various clinical studies have focused on the utilization of TAAs as an induction mechanism to trigger an immunologic response in patients with PCa [31,32].

### 4.1. Prostate Specific Antigen

PSA is composed of two beta-barrel domains and a kallikrein loop serin–protease structure that is solely expressed by epithelial cells. Murine studies have shown that PSA induces a specific T cell response. In a preclinical study, transgenic mice expressing human PSA were crossed with human leucocyte antigen (HLA)-A2.1-expressing mice to analyze the effects of androgen deprivation on T cells. The study results showed a substantial increase in cytotoxic lymphocytes specific to PSA, especially in the setting of androgen ablation [33].

### 4.2. Prostate Specific Membrane Antigen

PSMA is a transmembrane glycoprotein that has high specificity as a PCa cell surface ligand [34]. In addition, PSMA expression shows a correlative increase to higher-grade tumors and in the mCRPC setting [35]. The PSMA therapeutic potential has been explored as a potential candidate in target cell therapy and immunotherapy fields. Clinical trials in various settings have revealed that HLA-A2-restricted PSMA peptides elicit cytotoxic T cell lymphocyte (CTL) responses with antitumor characteristics in vitro [36,37,38,39], while in-vitro and xenograft studies have examined the possibility of PSMA as an immunotherapeutic molecule by studying the effects of antibodies targeting PSMA-expressing PCa tumor cells [40,41,42,43]. Recently, PSMA has been vigorously explored in various preclinical trials and clinical studies to enhance the T cell response via antigenic stimulation using genetically modified T cells that express chimeric anti-PSMA TCRs. In an ongoing phase II clinical trial conducted by De Giorgi et al., PSMA conjugated with 177 Lutetium (177 Lu-PSMA) is currently under analysis for its safety and efficacy as a potential treatment regimen for patients with mCRPC (NCT03454750) [44]. The VISION trial is a phase III clinical study assessing the clinical efficacy of 177 Lu-PSMA-617, a PSMA-targeting agent. The patient cohort consists of 750 patients with PSMA-positive PCa with a progressive disease with prior exposure to ARAT agents such as abiraterone or enzalutamide and taxane chemotherapy. The study arms are randomized to receive either 177 Lu-PSMA-617 with the best supportive/standard care or conservative management alone. Thus, the clinical trial aims to compare the OS rates between the two clinical cohorts (NCT03511664) [45].

### 4.3. Prostate Sten Cell Antigen

The PSCA is a cell surface protein anchored by glycosylphosphatidylinositol (GPI) and is known to be positively correlated with prostate cells and PCa in the advanced clinical setting and metastasis. Several studies have examined the viability of PSCA as a potential immunotherapy candidate by studying the tumor-specific CTL interaction generated by HLA-A2-restricted anti-PSCA peptides [46,47,48].

Moreover, this cell surface protein is being focused on as a novel approach for immunotherapy. According to various preclinical murine studies, conjugated PSCA antibodies and unconjugated forms of anti-PSCA antibodies have both shown antitumor properties toward PCa cells, which were observed in the cytotoxic reactions and regression of xenografts in mice [49,50,51]. Morgenroth et al. engineered CAR-T cells by transducing a CAR cassette specific to PSCA. The CAR-T cells showed significant activity in attacking PSCA-expressing cells, which show the potential immunologic effects of PSCA [52].

### 4.4. Prostatic Acid Phosphatase

PAP shows higher expression rates in intermediate-risk than high-risk PCa expressed in both benign and malignant prostate cells. Although PAP is considered as a TAA for PCa, this molecule is not technically a specific prostate antigen because it is expressed in other anatomical organs (e.g., the kidneys and testes) and in other solid tumor cells (e.g., breast, colon, and gastric cancer). In 2010, the study results of a phase III clinical trial showed that the administration of sipuleucel-T for the treatment of asymptomatic mCRPC or patients with minimal symptoms prolonged OS. The experimental arm cohort was exposed to APC that was activated ex vivo with PA 2024, a recombinant fusion protein that is constructed with a prostate antigen and PAP that is fused to G-CSF [53]. The sipuleucel-T arm showed a relative reduction in mortality risk of 22% compared with the control population in which the risk reduction denoted a 4.1-month improvement in median survival rates [53].

## 5. The Clinical Role of CAR-T Cell Therapy in the Setting of Metastatic PCa

Various clinical trials investigating the feasibility of CAR-T cell therapy in advanced or metastatic PCa have either been conducted or ongoing. However, while PSMA and PSCA are showing the most potential for future targets for CAR-T cell immunotherapy, other immunologic venues such as epithelial cell adhesion molecules (EpCAM) are being actively evaluated.

### 5.1. PSMA CAR-T Cells

Previous in-vitro and in-vivo clinical studies have proven that anti-PSMA CAR-T cells effectively target lesions or cells with PSMA expression [54,55] and provided a valuable rationale for the continuous evaluation of PSMA-targeting modalities for PCa management. Zuccolotto et al. studied the molecular interaction of PSMA CAR-T cells in the preclinical setting and revealed that the CAR-T cells effectively eradicated disseminated tumor cells after in-vivo transfer in tumor-bearing mice, which supported the use of this modality for clinical purposes [56]. In 2008, Junghans et al. initiated a phase I clinical trial studying the efficacy of first-generation anti-PSMA CAR-T cells (NCT00664196) [57]. However, study results were unsatisfactory due to the weak persistence of the CAR-T cells. In addition, only two of the five enrolled patients showed a partial response that was assessed by measuring the lowering of serum PSA levels [57]. Compared with the first generation, the modified second-generation CAR-T cells have higher clinical efficacy than its predecessor and represents a new immunotherapeutic approach in the ongoing battle against PCa in the advanced setting [58]. In a preclinical murine study, Ma et al. evaluated the therapeutic activity of a second-generation anti-PSMA CAR-T construct by inserting CD 28 as a costimulatory molecule [58]. Study results showed a significant decrease in tumor volume in mice injected with anti-PSMA CAR-T cells in comparison to nontransduced T cell-inoculated mice. Moreover, the therapeutic efficacy of a second-generation CD 28-based anti-PSMA CAR-T cell therapy was investigated (NCT01140373) in a phase I clinical trial lead by Slovin et al. [59]. Of the four enrolled patients, two showed stable disease. In the second cohort of the clinical trial, patients who received a higher dose of CAR-T showed a tolerable level of cytokine release syndrome (CRS). Thus, this study concluded that this particular approach is tolerable and that further studies are warranted to advance the molecular structure and engineering of CAR-T cells to increase efficacy [59]. Another clinical phase I trial (NCT03089203) is currently examining the feasibility of a combination of PSMA-specific and transforming growth factor-β (TGF-β)-resistant CAR-modified autologous T cells (CAR-T-PSMA-TGF-β RDN cells) [60]. These dual CAR-T cells have shown an increased survival rate and tumor lysis in mice models, which provides solid evidence for translation and use in the clinical setting.

The current status of immunotherapy on mCRPC has displayed limited treatment efficacy. Moreover, Zhang et al. developed a CAR-T cell therapy specific to PSMA, which was immune to the immunosuppressive qualities of TGF-β, to enhance the antitumor abilities of CAR-T cells. This was achieved by modifying the CD 8 T cells from patients with mCRPC with herpes simplex virus 1 (HSV-1) thymidine kinase [61]. Ganciclovir was used as a safety mechanism because the engineered CAR-T cell had HSV-1 thymidine kinase modified into the molecular construct. The study revealed that treatment with anti-PSMA CAR-T cells that were immune to TGF-β led to selective cell death of PSMA-expressing tumors. Moreover, tumor apoptosis increased infiltration of CD 8 cells along with an increase in interferon-gamma (IFN-γ), and IL-2 levels were only observed in PSMA-expressing cells [61]. In 2019, Hassani et al. assembled CAR-T cells against PSMA that was based on a camelid nanobody (VHH) construct [62]. This ultimately results in the increase of the therapeutic efficacy of CAR-T cells by circumventing the immunologic limitations caused by the fact that the majority of scFvs are based on mouse models and the immunogenicity of murine antigens in human subjects along with the relatively large scFv size. The specificity and therapeutic effects of VHH-CAR-T cells against PSMA-positive cells were established by the surge in IL-2 cytokine levels and a 38% increase in CD 69 expression [62]. Table 1 depicts current and ongoing clinical trials and studies based on PSMA CAR-T cell therapy.

### 5.2. PSCA CAR-T Cells

Metastatic PCa can be amenable to immunotherapy due to the wide expression of various tumor antigens, including PSCA. Priceman et al. showed that PSCA CAR-T cell costimulation determines the sensitivity of tumor antigen expression [63]. This study concluded that the 4-1BB costimulatory molecule improved selectivity for higher tumor antigen concentrations and tumor-killing ability, therefore proving the clinical significance of costimulation for the making of an optimal CAR-T cell therapy regimen after analyzing the costimulation activity of both CD 28 and 4-1BB [63]. An open-label clinical trial with a study cohort of patients with advanced prostate, pancreas, or stomach cancer with PSCA-expressing tumors is currently ongoing to analyze the safety and clinical properties of BPX-601, a PSCA-specific CAR-T cell (NCT02744287) [64]. The molecular properties of BPX-601 are controlled by a rimiducid-inducible MyD88/CD 40 costimulatory domain, which mediates antigen-independent CAR-T cell expansion and persistence that ultimately results in increased treatment efficacy [64]. Preliminary results of several cohorts of patients with pancreatic cancer revealed that the combination regimen of BPX-601 with a single dose of rimiducid was tolerable with mild adverse events and observed evidence of clinical benefit [65]. Table 2 summarizes ongoing clinical studies focused on PSCA CAR-T cell therapy.

### 5.3. EpCAM CAR-T Cells

EpCAM is a transmembrane glycoprotein expressed in various carcinomas, including PCa [66,67]. This molecule has a crucial role in cell signaling, migration, proliferation, and prevention of cell adhesion. EpCAM is found in prostate tumors and effusion and is expressed in patients with PCa with metastasis, making it a suitable target for the treatment of patients with PCa with advanced disease or metastasis. Thus, Deng et al. constructed an EpCAM-specific CAR and investigated its therapeutic abilities. The cells were used on two different cell lines: PC3 (the human PCa cell line with low expression rates of EpCAM) and PC3M (a metastatic clone of the PC3 cell line with EpCAM overexpression). Data from the study showed that this specific CAR-T cell had the therapeutic benefit not only in PC3M cells but also in prolonged survival rates in the PC3 prostate cell lines [68]. A phase I/II clinical trial (NCT03013712) is ongoing to evaluate the efficacy of EpCAM-specific CAR-T cells in solid tumors expressing EpCAM [69]. Table 3 shows ongoing clinical studies and trials based on EpCAM CAR-T cells.

## 6. Current Limitations of CAR-T Therapy in the PCa Treatment Setting

### 6.1. CAR-T Cell Persistence and Tumor Trafficking

CAR-T cell therapy has opened new possibilities as an innovative modality for PCa treatment. However, studies and clinical trials based on the therapeutic efficacy of CAR-T cells have shown unsatisfactory outcomes despite highly promising preclinical results. The efficacy of the T cell function is highly dependent on its migration abilities to the target tumor and its persistence rate. The persistence rate or survival period of CAR-T cell is a major determinant in the therapeutic efficiency of these modified T cells to target and destroy tumorous lesions. Although first-generation CARs have shown a meager persistence rate, higher persistent rates have been noted in the second- and third-generation CAR-T cells [30,70].

Prolonged persistence of CAR-T cells was traditionally attained by radiotherapy or chemotherapy. Current molecular research has shown that persistence can also be achieved by the administration of oncolytic viruses, which eradicates cells with suppressive properties including myeloid and regulatory T cells. Moreover, the immunosuppressive traits of the oncolytic viruses increase tumor immunogenicity and antigenicity, leading to a higher vulnerability of tumor cells to CAR-T cells and providing an environment suitable for vascular remodeling of the tumor lesions and the proliferation of adhesion molecules [71,72,73,74].

The homing properties of CAR-T cells to tumor cells or sites are known as trafficking, a mandatory prerequisite for T cell-induced tumor eradication. CAR-T cells and natural killer (NK) cells can be modified for better migration to the tumorous lesion through the additional expression of tumor-homing receptors [75,76,77,78], manipulation of bone marrow retargeting receptors such as CXCR 4 [79,80,81], or upregulation of CXCR 3 expression, which is linked to the inhibition of protein kinase A [82]. The aforementioned methods are experimental due to the participation of multiple immune-stimulatory cascades. Therefore, further prospective clinical trials are warranted to analyze the compatibility of these regimens to be recognized as a PCa treatment modality.

### 6.2. The Prostate Cancer Tumor Microenvironment

The unforgiving nature of TME exhibits another challenge for effective CAR-T cell therapy. After insertion into the tumor, CAR-T cells are surrounded by a harsh TME that has a low supply of nutrients and oxygen, low pH, and compact tumor stroma with residing immunosuppressive cells that excrete inhibitory molecules and increase inflammatory reactions. Moreover, the TME has a high concentration of reactive oxygen species (ROS), which hinders the antitumor activity of CAR-T cells [71]. In addition, Ligtenberg et al. investigated the effects of catalase coexpressed CAR-T cells and concluded that the introduction of a catalase to the cells mitigated the TME oxidative state with less accretion of ROS, and the catalase expressing CAR-T cell maintained its antitumor properties [83]. Mitigating the effects or neutralizing the TME provides a molecular challenge. Therefore, numerous methods for genetically modifying CAR-T cells or altering the microenvironment have commenced or are ongoing to address this subject.

### 6.3. Immune-Related Adverse Events

Although adoptive immunotherapy of CAR-T cell infusion has shown the treatment benefit for PCa in various clinical trials, the majority of treatments were associated with expected and unexpected toxicities and immune-related adverse events (IRAEs), which include CRS, tumor lysis syndrome, on-target/off-tumor toxicity, and neurological side effects [84]. Furthermore, CRS has been reported as one of the predominant side effects that patients experience after CAR-T cell infusion. The activation of infused autologous CAR-T cells stimulates cytokines that cause mild to severe inflammatory reactions and can be life-threatening in some cases [85]. The clinical features of CRS include systemic (e.g., fever, malaise, fatigue, myalgia, nausea, anorexia, tachycardia, hypotension, cardiac and renal impairments, and hepatic failure) and hematologic (e.g., disseminated intravascular coagulation) symptoms [84,86]. Other possible adverse reactions caused by CAR-T cell infusion is the on-target/off-tumor recognition. The autologous infusion of activated CAR-T cell can attack normal self-cells, which can ultimately result in autoimmune disease and organ damage because the target antigen is not solely restricted to the tumor and can be expressed in other cells and organs [87]. Various molecular approaches are currently under development or evaluated to further diminish the dangers of IRAEs. A promising method of overcoming probable toxic adverse events is the genetic modification and insertion of a suicide gene into CAR-T cells to deactivate the CAR-T cell in the event of a life-threatening reaction. A clinical study done by Di Stasi et al. established the role of caspase-9 in inducing T cell apoptosis [88]. Moreover, several studies proposed the insertion of a CAR vector into NK cells, which would substantially reduce the prevalence of IRAEs [89,90]. Other experimental approaches based on hematological malignancies and solid tumors including PCa [91] suggest that the solution to reducing immunologic side effects lies in the delivery of CAR-T cells using nanoparticles [92,93].

## 7. Conclusions

The field of ACT has opened new horizons in the treatment of patients with PCa with metastasis and advanced disease. CAR-T cell therapy for metastatic PCa has had a relatively short period of research and development and therefore has not shown significant results in the clinical field compared to other treatment regimens. Evidence accumulated in preclinical studies and early-phase clinical trials concludes that, due to the absence of a PCa-specific tumor antigen, genetic tailoring of PCa-specific T cells with more than one CAR cassette is needed for therapeutic T cells to persist and survive in the harsh TME surroundings. Moreover, further research is warranted for the increase of therapeutic efficiency of CAR-T cell therapy while reducing the risks of adverse events. Future clinical trials will provide the much-needed information for the engineering and design of the next-generation CAR-T cell that will be both tolerable and effective in patients with metastatic PCa. Although CAR-T cell therapy shows great potential as a viable treatment modality, this regimen has potentially life-threatening adverse events and safety concerns regarding its clinical applications. Future prospective studies are needed for the elucidation and better understanding of adoptive cell therapy for the physician to decisively assess the clinical implications and effectively implement CAR-T cell immunotherapy in metastatic PCa treatment.

## Figures and Tables

**Table 1:** Current and ongoing PSMA CAR-T therapy clinical studies and trials.

CAR-T Cell Type	CAR-T Cell Generation	Clinical Phase	Identifier or Study Title	Costimulatory Domain	Primary Endpoints
PSMA CAR-T	First generation	Preclinical	Targeted elimination of prostate cancer by genetically directed human T lymphocytes [54]		Tumor specificity Tumor response
PSMA CAR-T	Second generation	Preclinical	Human T lymphocyte cytotoxicity and proliferation directed by a single chimeric TCRζ/CD 28 receptor [55]	CD 28	Specific tumor lysis activity
PSMA CAR-T	Second generation	Preclinical	PSMA-specific CAR-engineered T cells eradicate disseminated prostate cancer in preclinical models [56]	CD 28	In vivo antitumor activity
PSMA CAR-T	Second generation	Phase I	NCT00664196 [57]		Safety partial response
PSMA CAR-T	Second generation	Preclinical	Advanced generation anti- prostate specific membrane antigen designer T cells for prostate cancer immunotherapy [58]	CD 28	PSMA-specific cytotoxicity
Autologous PSMA CAR-T	Second generation	Phase I	NCT01140373 [59]	CD 28	Safety, tolerability
Anti-PSMA TGF-β insensitive CAR-T	Second generation	Phase I	NCT03089203 [60]	4-1BB	Adverse events
Anti-PSMA TGF-β insensitive CAR-T	Second generation	Preclinical	Efficacy against human prostate cancer by prostate specific membrane antigen -specific transforming growth factor-β insensitive genetically targeted CD 8+ T cells derived from patients with Metastatic castrate-resistant disease [61]	4-1BB	Antitumor activity
VHH-CAR-T Anti-PSMA	Second generation	Preclinical	Construction of a chimeric antigen receptor bearing a nanobodyagainst prostate specific membrane antigen in prostate cancer [62]	CD 28	Cytotoxicity potential

CAR chimeric antigen receptor, PSMA prostate-specific membrane antigen, TGF-β transforming growth factor-β, VHH camelid nanobody.

**Table 2:** Current and ongoing PSCA CAR-T therapy clinical studies and trials.

CAR-T Cell Type	CAR-T Cell Generation	Clinical Phase	Identifier or Study Title	Costimulatory Domain	Primary Endpoints
PSCA CAR-T	Second generation	Preclinical	Costimulatory signaling determines tumor antigen sensitivity and persistence of CAR-T cells targeting PSCA+ metastatic prostate cancer [63]	CD 28, 4-1BB	Antitumor activity
BPX-601	Third generation	Phase I	NCT02744287 [64]	CD 40, MyD88	Toxicity, adverse events

CAR chimeric antigen receptor, PSCA prostate stem cell antigen.

**Table 3:** Current and ongoing EpCAM CAR-T cell therapy clinical studies and trials.

CAR-T Cell Type	CAR-T Cell Generation	Clinical Phase	Identifier or Study Title	Costimulatory Domain	Primary Endpoints
EpCAM CAR-T	Second generation	Preclinical	Adoptive T cell therapy of prostate cancer targeting the cancer stem cell antigen EpCAM [68]	CD 28	Antitumor activity
EpCAM CAR-T	Second generation	Phase I/II	NCT03013712 [69]	CD 28, CD 3	Toxicity Adverse events

CAR chimeric antigen receptor, EpCAM epithelial cell adhesion molecules.

## Data Availability

Data sharing is not applicable to this article.
